# The influences of age on olfaction: a review

**DOI:** 10.3389/fpsyg.2014.00020

**Published:** 2014-02-07

**Authors:** Richard L. Doty, Vidyulata Kamath

**Affiliations:** ^1^Department of Otorhinolaryngology: Head and Neck Surgery, Smell and Taste Center, Perelman School of Medicine, University of PennsylvaniaPhiladelphia, PA, USA; ^2^Division of Medical Psychology, Department of Psychiatry and Behavioral Sciences, The Johns Hopkins University School of MedicineBaltimore, MD, USA

**Keywords:** age, Alzheimer's disease, anatomy, olfaction, Parkinson's disease, physiology, psychophysics, neurodegeneration

## Abstract

Decreased olfactory function is very common in the older population, being present in over half of those between the ages of 65 and 80 years and in over three quarters of those over the age of 80 years. Such dysfunction significantly influences physical well-being and quality of life, nutrition, the enjoyment of food, as well as everyday safety. Indeed a disproportionate number of the elderly die in accident gas poisonings each year. As described in this review, multiple factors contribute to such age-related loss, including altered nasal engorgement, increased propensity for nasal disease, cumulative damage to the olfactory epithelium from viral and other environmental insults, decrements in mucosal metabolizing enzymes, ossification of cribriform plate foramina, loss of selectivity of receptor cells to odorants, changes in neurotransmitter and neuromodulator systems, and neuronal expression of aberrant proteins associated with neurodegenerative disease. It is now well established that decreased smell loss can be an early sign of such neurodegenerative diseases as Alzheimer's disease and sporadic Parkinson's disease. In this review we provide an overview of the anatomy and physiology of the aging olfactory system, how this system is clinically evaluated, and the multiple pathophysiological factors that are associated with its dysfunction.

## Introduction

The sense of smell determines our ability to perceive thousands of odors, including ones associated with such hazards as leaking natural gas, fire, and spoiled food. This important sense mediates, to a large degree, the flavor of foods and beverages and significantly enhances quality of life. We use this sense to confirm that our clothes, homes, and offices are clean, and to fully enjoy flowers, perfumes, festive occasions, personal care products, and nature (e.g., the mountains and the sea shore). It is perhaps not surprising, then, that smell loss or disordered smell function significantly impacts our safety, appetite, nutrition, and physical and mental well-being.

Cross-sectional studies suggest that about half of the United States population between 65 and 80 years of age has demonstrable smell loss and that, over the age of 80, approximately three-quarters experience such loss (Doty et al., 1984a; Duffy et al., 1995; Murphy et al., 2002). Somewhat lower prevalence estimates are seen in very healthy cohorts (Ship and Weiffenbach, 1993; Doty et al., 2011) and in some other populations (Bramerson et al., 2004; Karpa et al., 2010), although test methods and criteria for defining dysfunction vary considerably among studies. As a result of survivor bias and other factors, cross-sectional studies likely underestimate the prevalence of age-related olfactory dysfunction, and longitudinal studies are needed to determine incidence rates and individual changes that may occur over time from factors that damage the olfactory system (London et al., 2008). With rare exception (e.g., Schubert et al., 2011), few longitudinal studies have focused on olfactory function, *per se*, with most having the goal of detecting incipient dementia or other neurological disorders in older cohorts (Graves et al., 1999; Devanand et al., 2000; Wilson et al., 2007b; Herting et al., 2008; Olofsson et al., 2008, 2009; Ross et al., 2008; Schubert et al., 2008; Conti et al., 2013; Iranzo et al., 2013; Velayudhan et al., 2013). However, regardless of whether cross-sectional or longitudinal tests are employed, age-related decrements are robust and, as described later in this review, are detectable by any number of olfactory tests, including ones employing psychophysical, electrophysiological, and psychophysiological procedures. Results from most such tests are strongly correlated, reflecting, in large part, mutual dependence upon the integrity of common elements of the olfactory pathways.

The consequences of olfactory dysfunction are staggering. In addition to explaining why many older persons complain that food lacks flavor (Schiffman and Zervakis, 2002), decreased ability to smell is largely responsible for the disproportionate number of elderly who die in accidental gas poisonings and explosions each year. In Britain, ~10% of all accidental deaths in the home between 1931 and 1956 occurred from coal-gas poisoning, with the majority occurring in persons over the age of 60 years (Chalke et al., 1958). Stevens et al. have estimated that 45% of older adults are unable to detect petroleum gas diluted to the level dictated by safety standards, as compared to only 10% of younger adults (Stevens et al., 1987). In a 2004 study of 445 patients with chemosensory dysfunction, a number of whom were elderly, 37% of those with olfactory impairment reported having experienced an olfaction-related hazardous event at some point in their lives, as compared to only 19% of those with no such impairment. Cooking-related incidents were most common (45%), with ingestion of spoiled food (25%), lack of ability to detect leaking natural gas (23%), and inability to smell a fire (7%) being less frequent (Santos et al., 2004). One longitudinal study of 1162 older individuals without dementia found that the mortality risk was 36% higher in those with low scores on a smell test after adjusting for such variables as sex, age, and education (Wilson et al., 2011a).

This review addresses the functional and pathological changes that occur in the olfactory system as a result of age. The influences of age-related diseases on olfaction, such as Alzheimer's and Parkinson's disease, are briefly mentioned but not reviewed in detail; the reader is referred elsewhere for recent reviews of such influences (Barresi et al., 2012; Doty, 2012a,b). To provide a template for understanding the neural underpinnings of age-related changes in olfaction, we first provide an overview of basic olfactory anatomy and physiology. This is followed by the types of changes that are observed on a range of functional tests and their anatomical and neuropathological correlates.

## Basic anatomy and physiology of the nose and olfactory system

The human nose is comprised of two separate nasal passages separated by a septum. An external opening, termed the naris or nostril, enters into each chamber. Extending from the lateral walls of the chambers are nasal turbinates, cartilaginous structures that are covered with a highly vascularized epithelium which serves to warm, humidify, and cleanse the air (Frye, 2003). The latter is achieved by creating turbulent flow, largely as a result of a narrowing of the cavity close to the aperture of each naris (the nasal valve). The turbulence drives particulate and other airborne matter onto the nasal mucus which is continuously moved to the gut via respiratory cilia that beat in unison (Schwab and Zenkel, 1998). Damage to respiratory cilia is associated with bacterial build-up and other problems that can impair nasal function and, ultimately, the ability to smell (Cohen, 2006).

Before an odorant is able to initiate the neural activity responsible for smell perception, it must first enter the nasal cavity from either the nares or from the nasal pharynx (i.e., from the mouth) and be absorbed into the mucus covering the olfactory epithelium. This mucus, which differs in composition from that of the mucus within the nasal cavity proper, is largely derived from specialized Bowman's glands (Getchell and Getchell, 1992). Among its secretions are odorant-binding proteins that shepherd the transit of hydrophobic odorants to the olfactory receptors (Pelosi, 1994), growth factors associated with mitosis and other cell-related processes (Federico et al., 1999), numerous immune factors (Gladysheva et al., 1986), and biotransformation enzymes involved in not only odorant clearance, but in destruction of viruses and bacteria, degradation of pro-inflammatory peptides, and toxicant metabolism (Ding and Dahl, 2003).

**Figure 1:**
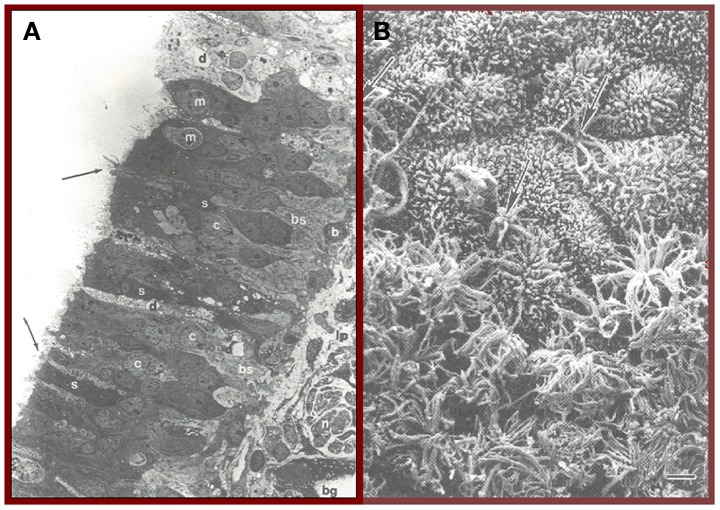
**(A)** Cross-section of the human olfactory epithelium. Four main types of cells can be discerned: bipolar receptor cells (arrows point to largely denuded cilia at dendritic knobs); c, cell body, microvillar cells (m), sustentacular cells (s), and basal cells (b); bg, Bowman's gland; lp, lamina propria; n, collection of axons within an ensheathing cell; d, degenerating cell; bs, basal cell undergoing mitosis. Photo courtesy of Dr. David Moran, Longmont, Colorado. **(B)** A transition zone between the human olfactory epithelium (bottom) and the respiratory epithelium (top). Arrows signify two examples of olfactory receptor cell dendrites with cilia that have been cut off. Bar = 5 μm. From Menco and Morrison (2003), with permission. Copyright©2003, Marcel Dekker, Inc.

The cells of the olfactory epithelium are derived embryologically from both the olfactory placode and the neural crest (Katoh et al., 2011). This specialized epithelium, which lines the upper regions of the septum, cribriform plate, superior turbinate, and sectors of the middle turbinate (Leopold et al., 2000), is innervated not only by olfactory receptor cells, but by fibers from the trigeminal nerve, the nervus terminalis (Cranial Nerve 0), and autonomic fibers from the superior cervical ganglion (Zielinski et al., 1989). In addition to the receptor cells, whose cilia differ from those elsewhere in the nose in lacking dynein arms and intrinsic motility, this epithelium is comprised of sustentacular (supporting) cells, microvillar cells, basal cells, and duct cells from the Bowman's glands (Menco and Morrison, 2003). Sustentacular cells add structural support to the epithelium and, among other things, insulate receptor cells from one another. These cells are also involved in the biotransformation of noxious chemicals and maintenance of the ionic milieu that surrounds the olfactory receptor cell cilia (Vogalis et al., 2005). Two morphologically distinct types of basal cells are recognized—electron-dense horizontal cells that express cytokeratin and electron-lucent globose cells which do not express cytokeratin (Mackay-Sim, 2010). It is from these multipotent stem cells, most notably the horizontal cells, that the olfactory receptor and other cell types germinate (Iwai et al., 2008). When damaged, the olfactory epithelium can be reconstituted from these cells, although the success of such regeneration is influenced by age-related processes such as telomere shortening (Watabe-Rudolph et al., 2011) and the degree of cumulative damage from prior environmental insults, including those from pollution and viral and bacterial infections (Harkema et al., 2006). The rate of mitosis of the basal cells is regulated by multiple processes, including local cell density, resident macrophages, neural activity, and damage to olfactory sensory neurons (Camara and Harding, 1984; Mackay-Sim and Patel, 1984; Borders et al., 2007). Biochemical and mechanical stress contribute to the sensory neuron differentiation from the basal cells (Feron et al., 1999). A cross-section of the olfactory epithelium is depicted in Figure 1A, whereas the ciliated surface is depicted in Figure 1B.

Over 350 different functional receptor proteins are expressed in the cilia of human olfactory receptor cells (Rouquier et al., 2000). Only one type of receptor protein is embedded in the ciliary membrane of a given receptor cell (Chess et al., 1994), even though most such cells respond to a range of odorant ligands (Holley et al., 1974; Sicard and Holley, 1984). Thus, the peripheral “olfactory code” is made up of activated sets of overlapping receptor cells that can be viewed as spatial maps within both the epithelium and the olfactory bulbs (Johnson and Leon, 2007). However, coding is complex, given that more types of receptors are recruited as an odorant's concentration is increased (Malnic et al., 1999; Kajiya et al., 2001). The olfactory receptors themselves are members of the heptahelical G-protein-coupled receptor (GPCR) superfamily whose genes are distributed across all but two chromosomes, with most being on chromosome 11 and the majority of the others on chromosomes 1, 6, and 9 (Glusman et al., 2001). Odorants bind to receptor pockets located on receptor transmembrane domains 3, 5, and 6 (Saito et al., 2009). The bond is not tight, with dwell times less than a millisecond (Bhandawat et al., 2005). Transduction results from activating a GTP-binding protein which, in turn, activates type III adenylyl cyclase, catalyzing the production of 3′,5′-cyclic monophosphate (cAMP) and opening cyclic nucleotide-gated channels. This results in the cellular influx of sodium and calcium ions and depolarization of the cell (Breer, 1994). Further amplification occurs from the opening of calcium-activated chloride channels and the resultant efflux of Cl^−^ from the cell (Stephan et al., 2009). While members of the trace amine-associated receptor (TAAR) family (Liberles and Buck, 2006) have been identified in the olfactory epithelia of a range of mammals, including humans, their role is poorly understood and ligands that activate murine TAARs do not activate intact primate orthologs (Staubert et al., 2010).

**Figure 2:**
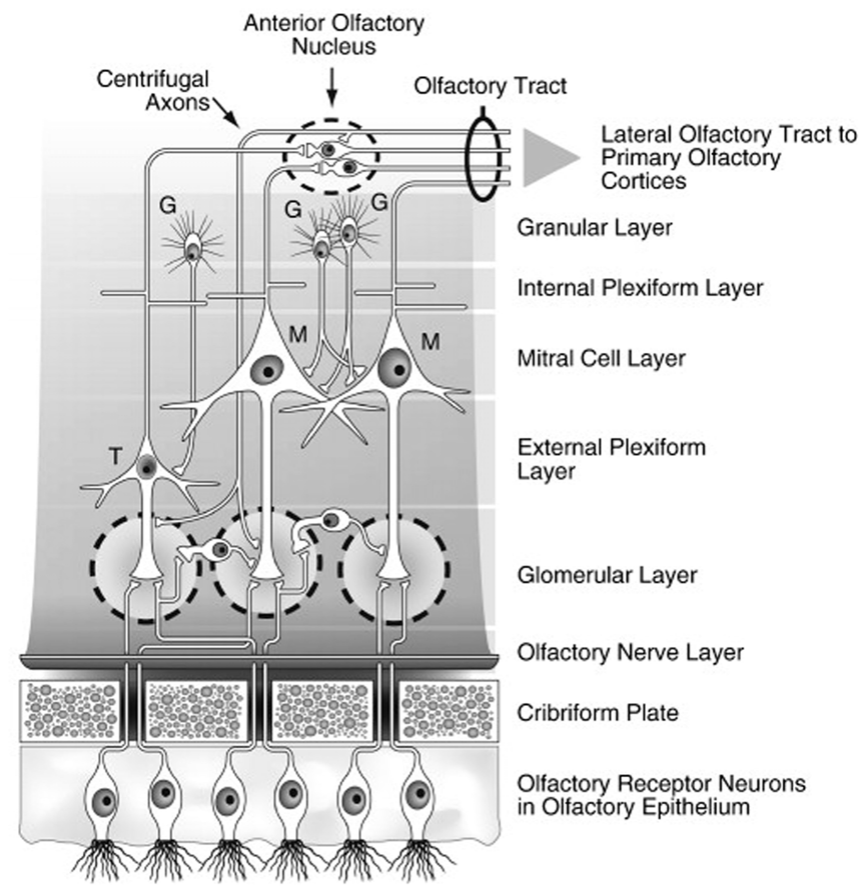
**Schematic drawing of the major layers of the olfactory bulb and the interactions between the different types of bulbar cells.** Abbreviations: G, granule cells; M, mitral cells; T, tufted cells. Note that the largely GABAergic granule cells send projections into the mitral cell and external plexiform layers, and that some small cells extend projections into more than one glomerulus. Reprinted with permission from Duda (2010), with permission. Copyright©2010, Elsevier B.V.

Bundles of olfactory receptor axons ultimately form the olfactory fila which are ensheathed by Schwann cell-like mesaxons, astrocytes, and fibroblasts. These bundles, which collectively make up Cranial Nerve I, aggregate beneath the basement membrane in the connective tissue-rich lamina propria and then penetrate multiple openings (foramina) of the cribriform plate—the thin sector of the ethmoid bone that separates the nasal cavity from the brain (De Lorenzo, 1957). The ensheathing cells have unique properties, as they not only provide guidance to axons projecting from the nasal cavity into the brain, but, along with monocytic cells, phagocytize bacteria and other xenobiotics which might otherwise enter into the brain (Smithson and Kawaja, 2010; Panni et al., 2013). Once inside the cranial cavity, the receptor cell axons make up the first of several layers of the olfactory bulb (Figure 2) and individually ramify into the globe-like glomeruli that constitute the next layer of the bulb (Meisami et al., 1998). In young persons, there are more than a thousand glomeruli, but this number decreases with age, reflecting, in part, loss of neurotrophic factors from degenerating receptor cells.

Interestingly, receptor cells that express the same receptor protein project to the same glomeruli, making the glomeruli, in one sense, functional representatives of the receptor types. The transmitter of the olfactory receptor cells, glutamate, acts upon NMDA and AMPA receptors on dendrites of projection neurons, the mitral and tufted cells. Juxtaglomerular cells modulate glutamate release via presynaptic D_1_ and GABA_B_ receptors on the receptor cell axon terminals (O'Connor and Jacob, 2008). Similar post-synaptic modulation occurs via activation of GABA and serotonin (5HT) receptors on the mitral and tufted cell apical dendrites. Additional modulation of these cells occurs within the bulb's external plexiform layer, where their secondary dendrites form, for example, dendro-dendritic connections with GABAergic granule cells, the most numerous cells of the olfactory bulb (Shepherd, 1972). Granule cell activity is modulated primarily by centrifugal input from neurons whose cell bodies fall outside of the olfactory bulb and which are influenced by central processes, including metabolic states. Importantly, some centrifugal neurons modulate the activity of microglial cells within the bulb and elsewhere which, if not kept in check, can induce nerve cell injury via the expression of Toll-like receptors that promote pro-inflammatory and pro-apoptotic activity (Lehnardt et al., 2007; Tang et al., 2007; Ziegler et al., 2007).

Like the olfactory neuroepithelium, a number of olfactory bulb cells, most notably periglomerular and granule cells, undergo periodic replacement (Bedard and Parent, 2004). There is evidence that considerable plasticity occurs within the glomerular region of the olfactory bulb throughout life, in addition to that which occurs during early post-natal development (LaMantia and Purves, 1989; Sawada et al., 2011). In humans, as in rodents and non-human primates, neural stem cells within the anterior portion of the subventricular zone (SVZ) of the brain generate neuroblasts even in adulthood. Some of these neuroblasts, in turn, migrate along the rostral migratory stream (a pathway extending from the SVZ to the olfactory bulb) to ultimately repopulate interneurons within the granule and glomerular layers of the bulb (Kam et al., 2009). There is some controversy, however, as the extent and nature of this migration in humans (Wang et al., 2011a).

The mitral and tufted cell axons project to more central olfactory structures, including the anterior olfactory nucleus, the piriform cortex, the rostral entorhinal cortex, and the corticobasal nuclei of the amygdala (Cleland and Linster, 2003). Since the afferent projections of the olfactory system to the cortex bypass the thalamus, some investigators have characterized the olfactory bulb as the “thalamus of the olfactory system” (Kay and Sherman, 2007). Although most bulbar projections to the aforementioned brain regions are ipsilateral, second order projections are made to the contralateral hemisphere via the anterior olfactory nucleus and anterior commissure. Subsequent connections are formed with the orbitofrontal cortex (OFC), hippocampus, thalamus, hypothalamus, and cerebellum (Cleland and Linster, 2003). The OFC, a multimodal structure, is thought to play a vital role in flavor perception, combining input from taste, texture, and smell (Rolls and Grabenhorst, 2008). Lesions in this area impair the identification of odors and flavors (Jones-Gotman and Zatorre, 1988). Because of these connections, one investigator has noted that “existing data suggest that [olfactory testing] may actually be among the most sensitive and selective measures of OFC dysfunction” (p. 464) (Zald, 2006).

## Age-related olfactory loss in humans and its quantification

Quantitative testing of the sense of smell, which is easy to perform in the clinic, is critical for identifying the nature and degree of smell dysfunction experienced by older persons. Many elderly fail to recognize their deficit or, when they do so, either overestimate or underestimate its magnitude. Importantly, a significant number complain of taste loss, not recognizing the primary contribution of olfaction in determining the flavor of their food. Based on quantitative testing, the clinician can inform many patients that their function, while diminished in an absolute sense, is still well above that of most of their peers, a point that provides considerable solace to those groping with the multiple changes that accompany the aging process.

Age-related deficits in olfactory function are detected by a number of types of olfactory tests, including psychophysical tests (e.g., tests of odor detection, identification, discrimination, memory, and suprathreshold intensity), electrophysiological tests (e.g., odor event-related potentials), and psychophysiological tests (e.g., odor-related changes in heart rate and respiration). All such tests generally detect age-related decrements in the olfactory system. Since hundreds of studies have documented such decrements, only selected examples are presented here.

### Psychophysical tests

Deficits observed in older persons have been most commonly detected using psychophysical tests—tests that require a conscious response on the part of the patient. With the possible exception of some measures of suprathreshold intensity and pleasantness, the results from the majority of psychophysical tests are positively correlated with one another (Doty et al., 1994; Koskinen et al., 2004), with the size of the correlations between any two tests being bounded by the reliability coefficient of the less reliable test (Doty et al., 1995). Thus, the weight of the evidence suggests that individuals have a “general olfactory acuity” factor similar to the general intelligence factor proposed for various tests of intelligence (Yoshida, 1984; Doty et al., 1994). Despite this evidence, and the fact that comparison of results from nominally disparate olfactory tests is confounded by differing reliabilities, odorants, non-olfactory task demands, and operational procedures, for heuristic reasons age-related deficits are described below for nominally distinct classes of tests. As will be seen, age-related effects are found regardless of the employed measuring instrument, although, in general, longer tests are more reliable and, hence, more sensitive to such deficits (Doty et al., 1995).

**Figure 3:**
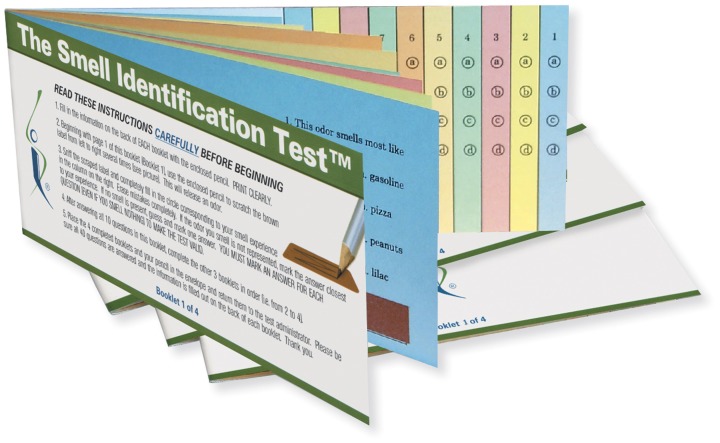
**The 40-item University of Pennsylvania Smell Identification Test (UPSIT).** This test is comprised of four booklets, each containing 10 microencapsulated (“scratch and sniff”) odors which are released by a pencil tip. The examinee is required to provide an answer on each test item (see columns on last page of each booklet) even if no odor is perceived or the perceived odor does not smell like one of the response alternatives (i.e., the test is forced-choice). This test has been administered to hundreds of thousands of subjects and is available in 15 different language versions. Photograph courtesy of Sensonics International, Haddon Heights, New Jersey USA. Copyright©2013, Sensonics International.

**Figure 4:**
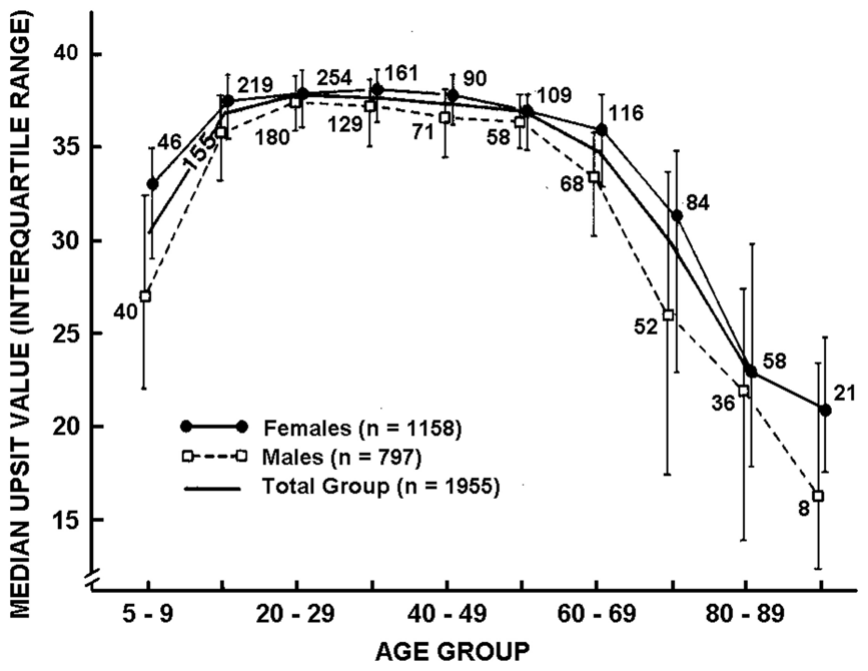
**Scores on the University of Pennsylvania Smell Identification Test (UPSIT) as a function of age and gender in a large heterogeneous group of subjects.** Numbers by data points indicate sample sizes. From Doty et al. (1984a), with permission. Copyright©1984, American Association for the Advancement of Science.

The most widely used psychophysical tests are *identification tests*. A number of identification tests, such as the 40-item University of Pennsylvania Smell Identification Test (UPSIT; Figure 3) and its briefer 12-item version (the Brief Smell Identification Test or B-SIT), can be self-administered. In such tests familiar odorants are presented and the subject is required to identify the name of the odor from written alternatives or, in some cases, to choose a picture that depicts the source of the odor (Doty et al., 1984b, 1996; Richman et al., 1992; Kobal et al., 1996; Hummel et al., 1997; Nordin et al., 2002; Kobayashi et al., 2006; Krantz et al., 2009; Cameron and Doty, 2013). In addition to absolute determination of function (e.g., normal or mild, moderate, severe, or total loss), sex- and age-related normative data are available for some such tests, making it possible to determine a patient's percentile rank relative to peers (Doty, 1995). Odor identification tests are clearly sensitive to age-related decrements in the ability to smell (Doty et al., 1984a, 2011; Cain and Stevens, 1989; Cain and Gent, 1991; Schiffman, 1991; Ship et al., 1996; Griep et al., 1997; Kaneda et al., 2000; Larsson et al., 2000; Murphy et al., 2002; Larsson et al., 2004; Calhoun-Haney and Murphy, 2005; Schumm et al., 2009; Hedner et al., 2010a,b; Olofsson et al., 2010; Wong et al., 2010; Schubert et al., 2011, 2012; Wehling et al., 2011; Wilson et al., 2011a; Menon et al., 2013; Pinto et al., 2013). Because a number of odors are not universally recognized, identification tests are often adjusted to contain odorants and response alternatives familiar to those in a given culture. An example of the prototypical age-related decrement present in odor identification is shown in Figure 4 (Doty et al., 1984a).

**Figure 5:**
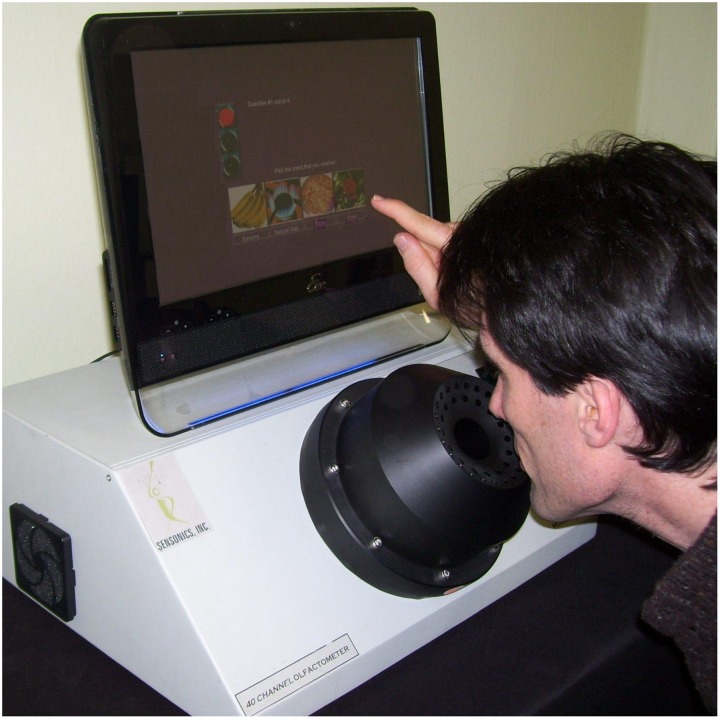
**The Self-administered Computerized Olfactory Testing System (SCOTS).** This modern olfactometer allows for self-administration of olfactory threshold tests, among other types of tests, and automatically calculates the threshold value based upon subject responses. This system eliminates administrator error in the presentation of test stimuli and provides exacting control of stimulus duration, inter-stimulus intervals, and other factors. Photograph courtesy of Sensonics International, Haddon Heights, New Jersey USA. Copyright©2013, Sensonics International.

Odor threshold tests are conceptually analogous to pure-tone hearing threshold tests, except that the stimuli consist of a range of concentrations of an odorant, rather than a range of tones. Unlike most auditory threshold tests, forced-choice testing is usually employed. In a given test, a series of different concentrations of an odorant are presented to a subject via sniff bottles, squeeze bottles, felt-tip pens, or olfactometers, such as the one depicted in Figure 5. Dilutions are commonly made in half-log steps using mineral oil, propylene glycol, or other liquids as the dilution media. The goal of the test is to detect the lowest odorant concentration that can be reliably detected (detection threshold) or recognized (recognition threshold) (Cain et al., 1983; Doty et al., 1984b; Takagi, 1989; Doty, 1995; Hummel et al., 1997).

As with odor identification tests, significant age-related alterations are generally observed regardless of the psychophysical paradigm used to establish the threshold (Chalke et al., 1958; Fordyce, 1961; Joyner, 1963; Kimbrell and Furchtgott, 1963; Venstrom and Amoore, 1968; Strauss, 1970; Schiffman et al., 1976; Murphy, 1983; Van Toller and Dodd, 1987; Cain and Gent, 1991; Stuck et al., 2006; Larsson et al., 2009), with somewhat lower thresholds (greater sensitivity) in the healthiest cohorts (Griep et al., 1997). Studies that have explored a spectrum of ages typically report age-related performance functions similar to those depicted for odor identification (Figure 3), although such functions depend upon the involved odorant (Venstrom and Amoore, 1968; Deems and Doty, 1987).

Suprathreshold odor discrimination tests require the subject to discriminate among sets of odorants or odorant mixtures, for example by identifying the “odd” stimulus or set of stimuli from foils (Jehl et al., 1995; Kobal et al., 2000; Weierstall and Pause, 2012). In some instances, similarities among odorants are established and the similarity ratings or correlations subjected to statistical procedures such as multidimensional scaling, a procedure that aids in visualizing how well the stimuli can be differentiated from one another (Schiffman and Leffingwell, 1981). Older persons, on average, are less able than younger ones to discriminate between stimuli (Schiffman and Pasternak, 1979). Some match-to-sample discrimination tests intersperse differing delay intervals between the inspection odor and response set (Bromley and Doty, 1995; Choudhury et al., 2003). The goal is to assess the ability to remember the inspection odor and chose it from foils. However, the odor memory component of such tests can be confounded with semantic issues (e.g., labeling an odor with a name and then remembering the name of the odor whose memory is already present in long-term memory stores) (Jonsson et al., 2011). Such confounding can be overcome to some degree by using unfamiliar odorants that are difficult to consistently label or by employing incidental memory tasks (Møller et al., 2004, 2007).

**Figure 6:**
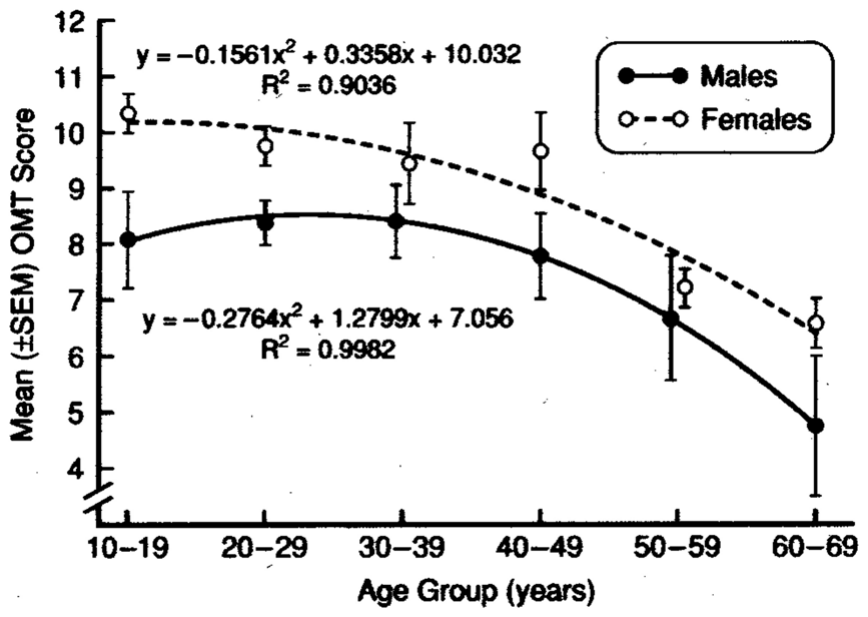
**Test scores for men and women on a 12-item odor discrimination/memory test as a function of age.** Note age-related decline in performance and the fact that women outperform men at all ages. Data are collapsed over 0-, 30- and 60-s delay intervals. From Choudhury et al. (2003), with permission. Copyright©2003, Oxford University Press.

An example of the influences of age and sex on an odor discrimination/memory test is presented in Figure 6. In this study, no effects of 10, 30, and 60 s delay intervals were observed, supporting the view that short-term odor memory is not affected in most persons and that performance differences in match-to-sample tests largely reflect discrimination, *per se* (Engen et al., 1973; Choudhury et al., 2003). It should be emphasized that it is probably impossible to completely disassociate memory processes from other nominal forms of odor perception, since memory is involved in most olfactory tasks and consciousness itself is, in effect, a form of memory. Importantly, age-related deficits in odor recognition may be a reflection of greater difficulties in recalling odor knowledge or names than in poorer ability to perceive or recognize the involved odors (Larsson et al., 2006).

Suprathreshold measures of the perceived strength of odors, as assessed using rating scales and magnitude estimates, have been shown to be sensitive to age in some, but not all, studies. Differences in procedures, odorants assessed, and sample sizes likely explain such discrepancies. In a study of over 26,000 respondents to a scratch-and-sniff odor survey of members of the National Geographic Society, ratings of the strength of single concentrations of six odorants were obtained using a 5-point rating scale (Wysocki and Gilbert, 1989). Age-related declines in the ratings were most noticeable for mercaptans (26% decline over the life span) and amyl acetate (22% decline), with less decline occurring for eugenol (14%), rose (13%), androstenone (10%), and Galaxolide (3%). Those odorants that showed the least decline were initially rated as less intense and were usually more difficult for older persons to identify. Importantly, when the data from the six stimuli were averaged, the age-related declines in the odor ratings began for males in their 20's and for females in their 40's.

**Figure 7:**
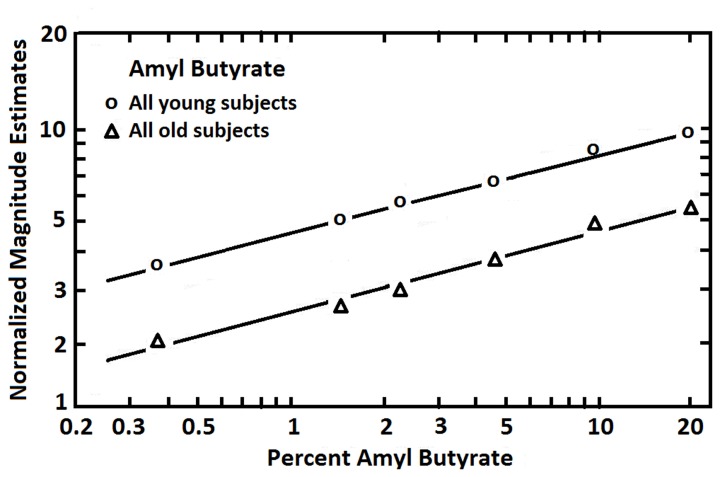
**Magnitude estimates given to six concentrations of amyl butyrate after adjustment for number usage by the employment of a cross-modal matching procedure.** Each age group was comprised of 10 men and 10 women. The younger group ranged in age from 18 to 25 years, and the older group from 65 to 85 years. From Stevens et al. (1982), with permission. Copyright©1982, ANKHO International, Inc.

Findings from studies assessing age-related changes in perceived intensities across multiple suprathreshold odorant concentrations have been variable. Most studies have employed magnitude estimation procedures to assess the build-up of stimulus intensity as odorant concentration is increased. In the classical magnitude estimation procedure, subjects are instructed to assign numbers in proportion to the relative perceived intensity of different concentrations of an odor (e.g., if a stimulus smells twice as strong as another, a number twice as large is assigned, and so on) (Doty and Laing, 2003). Each subject is allowed, in most instances, to choose the specific numbers they wish to employ (the “free modulus” method). In some cases, responses other than numbers are used, such as pulling a tape measure a distance proportional to the perceived intensity. Murphy (1983), using the mixed olfactory/trigeminal stimulant menthol, found the slope of the stimulus:response magnitude estimation function of 10 older persons to be less steep than that of 10 younger persons. However, other investigators have not observed such stark slope differences. In a study of 120 subjects ranging from 6 to 94 years of age, for example, magnitude estimates made to various concentrations of 1-propanol were unrelated to age, leading the authors to erroneously conclude that age did not influence olfaction (Rovee et al., 1975). Similarly unimpressive age-related effects were observed in a study of 137 subjects that assessed the intensities of phenyl ethyl alcohol and pyridine (Cowart, 1989). Stevens et al., in a study of 20 young and 20 old subjects, also found no strong evidence for meaningful age-related altered slopes in stimulus:response functions for amyl butyrate, a relatively non-pungent odorant, or CO_2_, a strong trigeminal stimulant (Stevens et al., 1982). However, by using the method of cross-modal matching, the relative position of parallel stimulus:response functions was found to differ between the young and old subjects (Figure 7). In this procedure, low pitch broad-band tones were interspersed among the odorant concentration trials and the subjects were required to estimate the intensities of both the tones and the smells relative to one another. Under the assumption that the broad-band low frequency tones were not markedly influenced by age, differences in idiosyncratic number usage could be taken into account, allowing an assessment of the magnitude of absolute odor intensity estimates. As is clear from Figure 5, the percentage decrement in strength observed in older subjects was uniform across concentrations. Similar findings have been noted in cross-modal matching studies for amyl acetate, amyl butyrate, benzaldehyde, ethyl alcohol, limonene, and pyridine (Stevens and Cain, 1985).

### Electrophysiological tests

Odor-induced recordings have been obtained from electrodes placed near or on the olfactory epithelium, producing a summated negative potential termed the electro-olfactogram (EOG) (Hosoya and Yashida, 1937; Ottoson, 1956). EOG magnitude is proportional to stimulus concentration and is correlated with perceived intensity, although it can be present even after death, suggesting it alone cannot be relied upon as a measure of odor perception, *per se*. Recording can be tedious and activity in one area of the olfactory epithelium is not necessarily representative of activity in other areas. Although one can surmise from the age-related general decreases in the integrity of the olfactory epithelium that EOGs would be expected to be smaller, to our knowledge no such study has been performed. It is noteworthy, however, that EOG activity is found on the anterior surface of the middle turbinate and a few millimeters below the anterior insertion of the middle turbinate, suggesting, along with biopsy samples, that the olfactory epithelium extends farther forward in some people than traditionally believed (Leopold et al., 2000).

**Figure 8:**
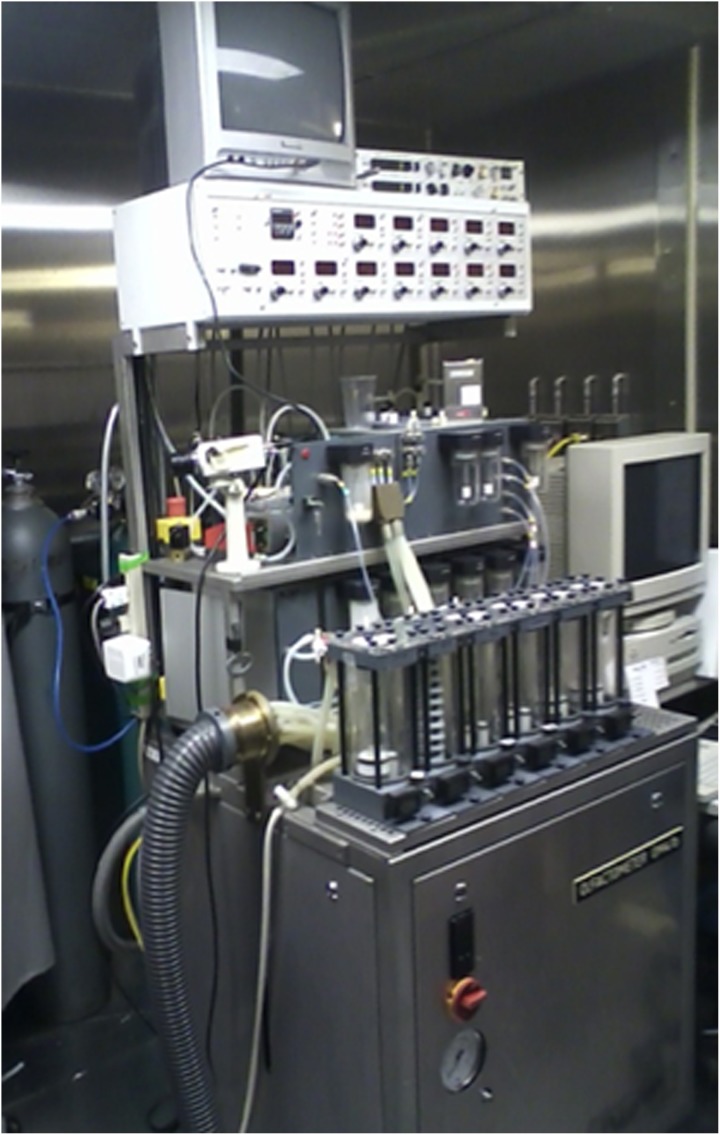
**Air-dilution olfactometer used to present pulses of odorants into a purified and humidified airstream directed through nares of a subject.** This device ensures that the odor event-related potentials (OERPs) are not confounded by somatosensory artifacts due to alterations in stimulus pressure, temperature, or other factors. Photo courtesy of the University of Pennsylvania Smell and Taste Center, Philadelphia, PA.

**Figure 9:**
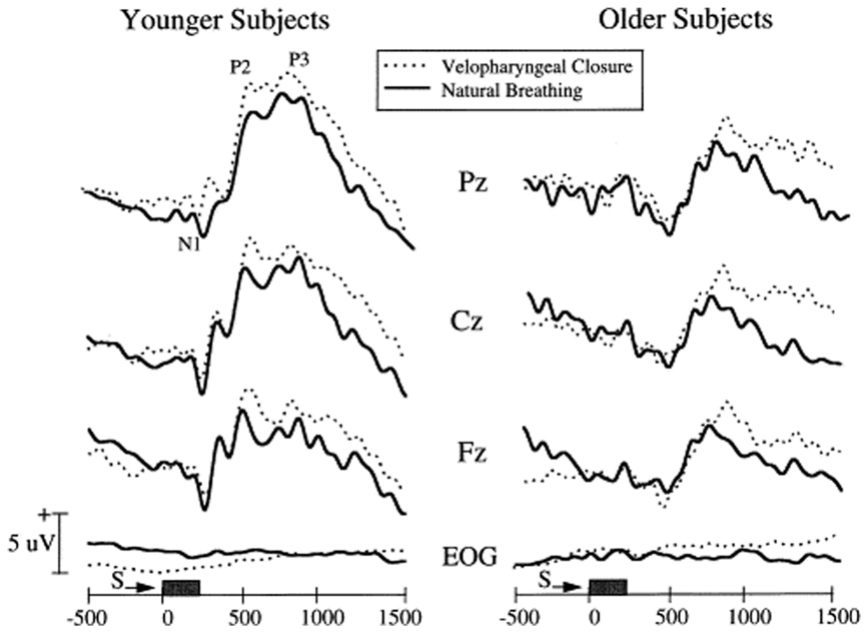
**Olfactory event-related potentials obtained from 12 younger (mean age: 24 years) and 12 older (mean age: 71 years) subjects using normal breathing or breathing after being trained to close the palate to minimize airflow from the mouth (velopharyngeal closure).** Note the smaller amplitude and longer latency responses in the older group. From Thesen and Murphy (2001), with permission. Copyright©2001, Elsevier Science B.V.

A more practical electrophysiological procedure is the measurement of odor-induced electrical activity at the level of the scalp (e.g., the odor event-related potential or OERP). This activity reflects odor-related changes induced in electrical fields generated by large populations of cortical neurons (Gevins and Remond, 1987). However, the signals are small (<50 μV), can be difficult extract from the background EEG, and require complex stimulus presentation equipment (Figure 8). In one study, for example, OERPs were not identifiable in nearly a third of subjects with no olfactory deficits (Lotsch and Hummel, 2006). Nevertheless, age-related alterations in the latency and amplitude of OERPs have been observed, with older persons typically exhibiting longer N1 latencies and smaller N1 and P2 amplitudes (Figure 9) (Murphy et al., 1994; Evans et al., 1995; Hummel et al., 1998; Covington et al., 1999; Thesen and Murphy, 2001; Stuck et al., 2006; Morgan and Murphy, 2010). Although classic procedures analyze only time-locked potentials, recently developed procedures combine traditional EEG and OERP methodology to assess activity within both time and frequency domains (Huart et al., 2012; Osman and Silas, 2014). Age-related responses using these newer methods have yet to be assessed.

### Psychophysiological tests

Psychophysiological tests are tests which measure mainly autonomic nervous system responses to stimuli, in this case odor. Among such measures are changes in heart rate (Bensafi et al., 2002), blood pressure (Nagai et al., 2000), respiration (Kleemann et al., 2009), and skin conductance (Møller and Dijksterhuis, 2003). In the case of the nose, cardiovascular and respiratory changes can reflect activity of the trigeminal nerve (CN V), rather than the olfactory nerve (CN I), limiting in some cases the usefulness of such tests (Allen, 1928).

**Figure 10:**
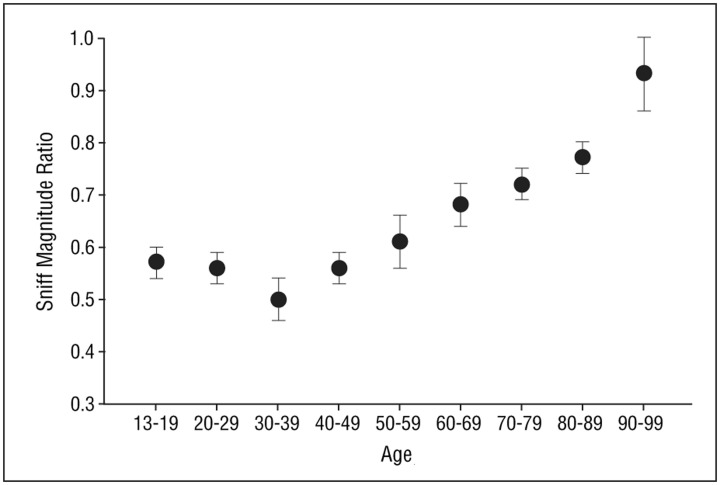
**Mean (s.e.m.) sniff magnitude ratios obtained from the Sniff Magnitude Test as a function of age.** Sample size = 137 subjects, 74% of whom were female. From Frank et al. (2006), with permission. Copyright©2006, American Medical Association.

A recently developed test measures a basic respiratory response to smelling an unpleasant odor (Frank et al., 2003, 2004, 2006). In this test, called the Sniff Magnitude Test, the subject sniffs a canister. Upon the initiation of the sniff, which is detected by air pressure changes sensed by cannulas positioned just inside the nose, the canister opens and either odorless air or a bad smelling odorant (e.g., methylthiobutyrate or ethyl 3-mercaptoproprionate) is released. Persons with a good sense of smell immediately stop sniffing, whereas those with a poor sense of smell take longer to inhibit their sniff or, if anosmic, may not inhibit their inhalation at all (Tourbier and Doty, 2007). The magnitude and duration of the sniff is assessed by a computer and a ratio computed between the area of the sniff pressure-time curve on odorant trials to that on blank air trials. Like other olfactory measures, this measure is sensitive to age-related olfactory changes (Figure 10) (Frank et al., 2006).

## Causes of age-related olfactory loss

It seems intuitive that structural changes would be present in the aging nose and olfactory system that would explain the functional declines observed in older persons. Indeed, as described below, a number of age-related alterations within the nose, olfactory epithelium, bulb, and higher brain structures have been associated to one degree or another with olfactory dysfunction. Moreover, several genes have been found to contribute, albeit to a modest degree, to the age-related decline in odor identification. For example, persons over the age of 70 who are homozygous for the val allele of the val66met polymorphism of brain derived neurotrohic factor (BDNF) exhibit a somewhat greater 5-year decline in odor identification performance than persons heterozygous for this allel (v/m) or homozygous for the met allel (m/m) (Hedner et al., 2010b). Older carriers of the ε4-allele of the human apolipoprotein E gene, a plasma protein involved in lipid transport, exhibit greater longitudinal declines in odor identification than non-carriers (Calhoun-Haney and Murphy, 2005). This occurs even after controlling for the effects of vocabulary and general cognitive status, suggesting that the influences of this allele on odor identification ability are independent of clinical dementia (Olofsson et al., 2010).

More recently, Doty et al. tested the odor identification ability of 1222 very old twins and singletons, including 91 centenarians (Doty et al., 2011). Unlike cognition, the sex- and age-adjusted heritability coefficients for odor identification from the two genetic models employed were quite low (i.e., 0.13 and 0.16 compared to 0.70 for cognition). These authors point out that nearly all twin studies looking at middle aged or older study cohorts report low heritability coefficients, in contrast to studies in which only young cohorts are assessed. One explanation for this observation is that the initial effects of heritability on function are eventually swamped by other factors in older persons, including the cumulative environmental insults to the olfactory epithelium, as described below.

### Changes in non-olfactory elements of the nose

Odorant access to the olfactory receptors can be altered by age-related changes in nasal airflow patterns and mucous composition, including those associated with diseases which are more common in the elderly. The prevalence of chronic rhinosinusitis, nasal polyposis, and lessened mucocilliary clearance increases with age (Settipane, 1996; Cho et al., 2012), as does nasal resistance, as measured by rhinomanometry (Edelstein, 1996). The nasal epithelium undergoes age-related atrophy, decreases in mucosal blood flow, and decrements in elasticity (Somlyo and Somlyo, 1968; Bende, 1983). In general, older persons report experiencing more frequent episodes of postnasal drip, nasal drainage, sneezing, and coughing than younger ones (Edelstein, 1996). Interestingly, increased age is associated with a significant *decrease* in asthma (Jarvis et al., 2012) and a number of abnormalities of the nasopharynx, such as adenoidal hypertrophy, inflammation, cystic degeneration, or thick mucus discharge (Edelstein, 1996). Recently it has been shown that sleep apnea, a disorder associated with restriction of nasal airflow that increases in prevalence with age, has an adverse effect on smell function (Salihoglu et al., 2013).

It must not be forgotten that the nose is a dynamic organ. Airflow patterns are regularly shifting, reflecting multiple influences on nasal turbinate engorgement and secretory activity from air temperature, humidity, physical activity, psychological stress, and environmental xenobiotics such as allergens, nanoparticles, toxic chemicals, and infectious agents (Frye, 2003). Nasal engorgement is regulated in large degree by the autonomic nervous system. Thus, relative sympathetic/parasympathetic dominance influences the lateralized changes in engorgement of the nasal capillary bodies that occur over time. Although *reciprocal* and *cyclic* left:right fluctuations in relative airflow—termed the nasal cycle—are not as common as previously believed (Gilbert, 1989; Mirza et al., 1997), there is evidence that olfactory sensitivity is somewhat higher during the so-called sympathetic phase of this putative cycle, i.e., when the left side of the nose is relatively more engorged than the right (Frye and Doty, 1992). Since nearly three-quarters of adults over the age of 50 no longer exhibit this cycle (Mirza et al., 1997), a subtle lowering of olfactory sensitivity could mark the transition from the predominance of relative more sympathetic dominance to a more balanced sympathetic/parasympathetic mode. Age-related changes the suprachiasmatic nucleus, a brain center involved in the control of a number of biological rhythms, could conceivably account for this effect (Farajnia et al., 2014).

**Figure 11:**
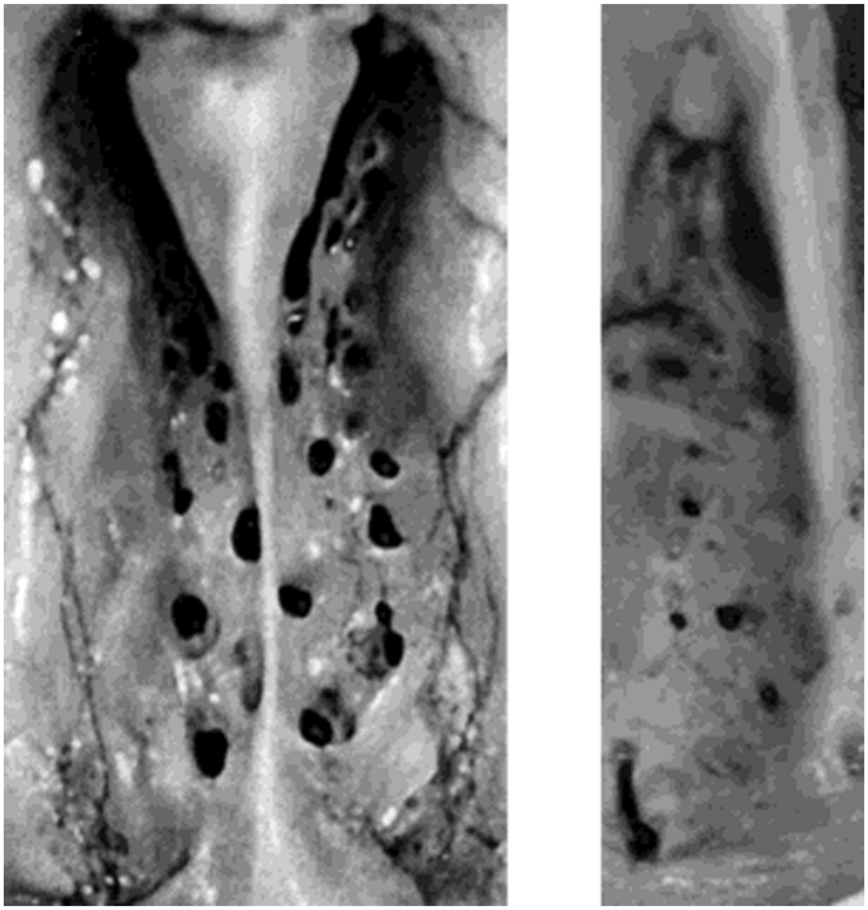
**Left:** left and right halves of the cribriform plate of a 25-year-old female in superior view. **Right:** left half of cribriform plate of a 66-year-old male in superior view. Note the difference in size and number of patent foramina that transmit cranial nerve I between the young and old cribriform plates. Anterior is toward top. From Kalmey et al. (1998), with permission. Copyright©1998, Wiley-Liss, Inc.

An important non-neural process that undoubtedly compromises smell function is the age-related decline in the size and number of patent foramina of the cribriform plate (Krmpotic-Nemanic, 1969; Kalmey et al., 1998). The occlusion or decrement in size of these holes can lead to a pinching off or elimination of olfactory receptor cell axons that enter into the brain from the olfactory epithelium (Figure 11). Kalmey et al. found a 47.3% reduction of the area of the foramina within the posterior centimeter of the cribriform plate in men older than 50 years relative to those younger than this age (7.19 vs. 3.79 mm^2^) (Kalmey et al., 1998). The reduction in women was 28.8% (5.61 vs. 3.99 mm^2^).

As described in the next section, the olfactory neuroepithelium becomes compromised as we age. While there are multiple reasons for this compromise, it is clear that the clearance of bacteria and other agents from the nasal cavity—clearance that depends in large part on the nature of the mucus and mucocilliary activity—changes across the lifespan. In one study of adults ranging in age from 18 to 100 years, for example, mucociliary function was found to be impaired in about 30% of those 60 years of age and older (Sakakura et al., 1983).

### Changes in the olfactory neuroepithelium

**Figure 12:**
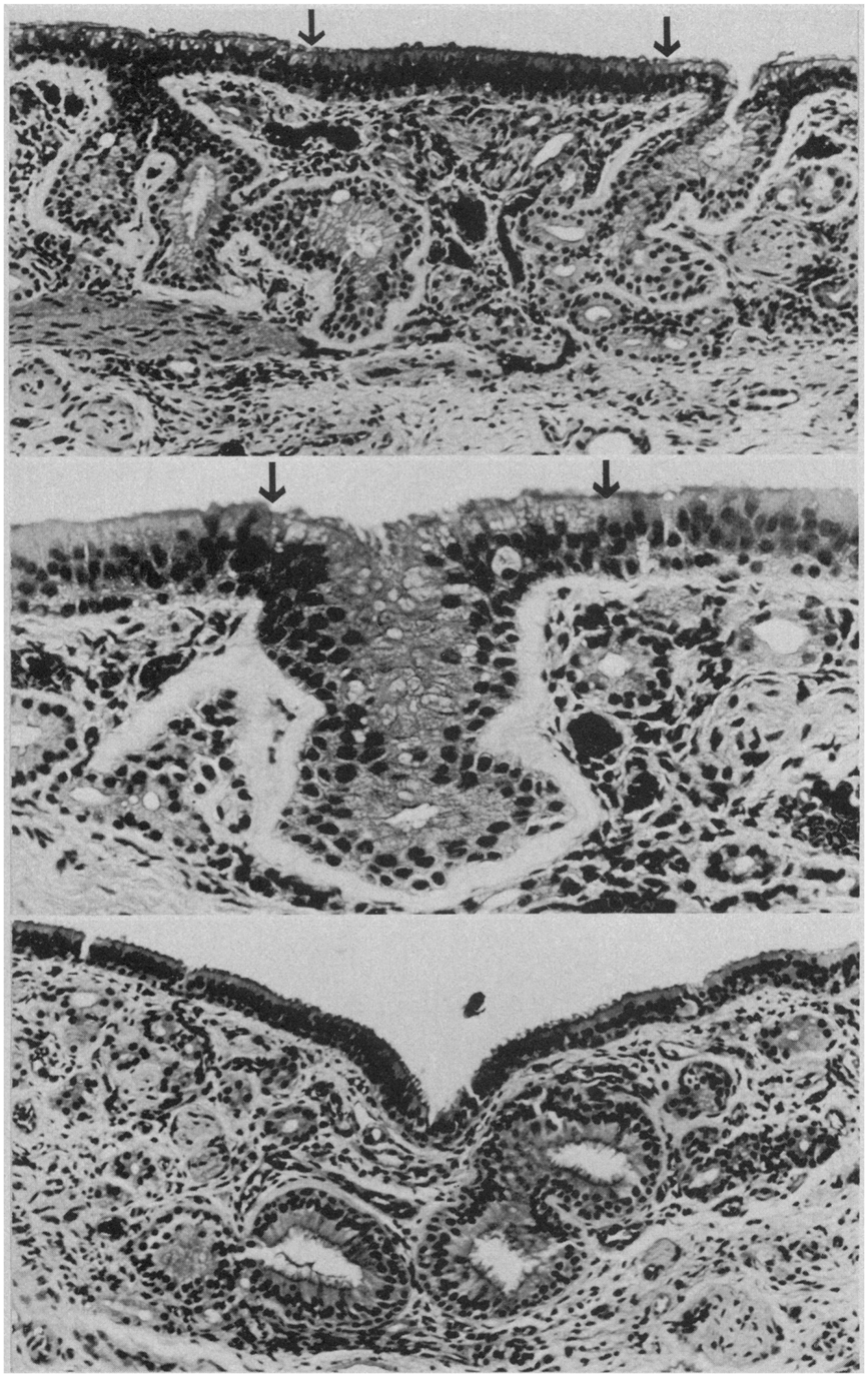
**Respiratory epithelium in the olfactory region of the adult human. Top:** ciliated and goblet cell-containing respiratory epithelium has invaded degenerated olfactory neuroepithelium (between arrows). Arrows indicate junction of respiratory and olfactory epithelia (HandE, × 100). **Middle:** gland-like invagination (between arrows) of respiratory epithelium into the lamina propria (HandH, × 200). **Bottom:** gland-like respiratory epithelium with large lumina in the lamina propria (HandE, × 1000). From Nakashima et al. (1984), with permission. Copyright©1984, American Medical Association.

Histological studies of the human olfactory epithelium have shown age-related changes in its nature and integrity, including decreased number of receptors, a thinning of the epithelium in general, and alterations in the cellular patterns and zonal distributions of the nuclei of the olfactory receptor and sustentacular cells. Intermingling of supporting and receptor cell nuclei are common, as is intercalation of respiratory epithelium with that of the olfactory epithelium, reflecting replacement of the olfactory epithelia with respiratory epithelia (Naessen, 1971; Nakashima et al., 1984; Morrison and Costanzo, 1990; Paik et al., 1992) (Figure 12). Similar alterations are noted in rodents exposed to olfactory toxins such as 3-methyl indole and 3-trifluoromethyl pyridine (Gaskell et al., 1990; Peele et al., 1991). In infancy and early childhood, the olfactory epithelium is highly vascularized, with blood capillaries being found in its basal layers and in close association with the perikarya of the receptor cells (Naessen, 1971). With age, these intraepithelial vessels regress and the epithelium becomes avascular. In adulthood, pigment granules are evident in the cytoplasm of sustentacular cells—granules that increase in number in the elderly (Naessen, 1971).

There are a number of reasons for the age-related decline in olfactory receptor cells and other elements of the olfactory epithelium. *First*, neurogenic processes appear to be compromised with age. In the rat, the ratio of dead or dying cells to the number of live receptor cells increases with aging (Mackay-Sim, 2003), suggesting the possibility that receptor cells from older individuals have less mitotic activity than those from younger individuals. This is in accord with the observation that following chemical destruction of the olfactory receptors of mice with zinc sulfate or methyl-formimino-methyl ester, morphological repair is slower or nonexistent in older animals (Matulionis, 1982; Rehn et al., 1986). *Second*, the aforementioned age-related decline in the size and number of patent foramina of the cribriform plate may result in necrosis of the olfactory receptor cells, eliminating them from the olfactory epithelium (Krmpotic-Nemanic, 1969; Kalmey et al., 1998). *Third*, immunologic and enzymatic defense mechanisms critical for maintaining the integrity of the epithelium become compromised with age. For example, age-related reductions have been found in the expression of phase I and phase II xenobiotic metabolizing enzymes, including carnosinase, glutathione, S-transferases, heat-shock protein 70, and isoforms of cytochrome P-450 (Kirstein et al., 1991; Getchell et al., 1995; Krishna et al., 1995). *Fourth*, age-related losses occur in the specificity of the responses of individual receptor cells. For example, electrophysiological tuning curves are broader in biopsied receptor cells from older than from younger persons (Rawson et al., 1998). *Fifth*, exposures to air-borne environmental agents, including air pollution, cigarette smoke, viruses, bacteria, and other xenobiotics, damage regions of the olfactory epithelium, having more functional consequence in later years when their cumulative effects have taken a toll on the epithelium (Smith, 1942; Hirai et al., 1996; Loo et al., 1996). As mentioned earlier, environmental factors likely swamp age-related genetic factors in determining the degree of olfactory function in later life (Doty et al., 2011). Thus, Loo et al. found no age-related decrement in the number of mature olfactory neurons in the olfactory mucosa in rats reared in a pathogen-free environment, unlike the situation in rats reared in a normal laboratory environment (Loo et al., 1996).

### Changes in the olfactory bulb

In parallel with the integrity of the olfactory epithelium, the size of the olfactory bulb and a number of its laminae—most notably the glomerular layer—declines with age in humans and other animals (Bhatnagar et al., 1987; Yousem et al., 1998; Sama et al., 2008). While this decline may reflect, to some degree, generalized atrophy, loss of neuronal elements, and increases in astroglia, most of the decline appears to be secondary to damage to the olfactory neuroepithelium from nasal infections, chronic rhinitis, lack of airflow, and exposures to xenobiotics; (see Holt, 1917; Frühwald, 1935; Smith, 1935; Meurman, 1950; Liss and Gomez, 1958). Indeed, one can use the number of glomeruli of autopsy specimens to infer the amount of destruction of the olfactory epithelium. In a classic study, Smith (1942) did just that, counting the number of glomeruli to estimate age-related losses of human olfactory receptors. In his examination of 205 olfactory bulbs from 121 autopsy specimens, he concluded that the loss of the olfactory nerves begins soon after birth and continues throughout life at the rate of approximately 1% per year. However, considerable variability was noted at all ages and a reassessment of his data using medians rather than means suggests that glomerular loss is most evident after the fifth decade of life, more or less paralleling what is shown in Figure 3 for odor identification.

Bhatnagar et al. (1987) quantitatively assessed the morphology of eight pairs of bulbs from women who died between the ages of 25 and 102 years. Corresponding bulb volumes, estimated for the ages of 25, 60, and 95 years by linear regression, were 50.02, 43.35, and 36.68 mm^3^. Mean mitral cell numbers for these three age groups were 50,935, 32,718, and 14,501, respectively. Other cell types were not enumerated. As would be expected from the work of Smith (1942), the glomerular layer was markedly influenced by age and, in the older specimens, was discontinuous and evident only in the rostral areas of the bulb.

Some studies have noted, in brains from non-demented older persons, neurofibrillary tangles in the olfactory bulb that increase as a function of age. For example, Kishikawa et al. (1990) observed such tangles in 35.3% of olfactory bulbs from 133 individuals ranging from 40 to 91 years (mean = 64.3 years), only one of whom had dementia. Most of the tangles were within the anterior olfactory nucleus, although a few were found in mitral and tufted cells. This percentage increased to 40.5% when only those over the age of 50 years were included in the sample. Similar types of pathology have been noted in autopsied olfactory bulbs from young persons who had lived in highly polluted regions of Mexico City, in some cases in association with nanoparticles that have entered into the bulbs via the olfactory fila (Calderon-Garciduenas et al., 2010).

In the first of a series of important quantitative studies, Hinds and McNelly measured the volume of the glomerular, external plexiform, internal plexiform, and olfactory nerve layers of the olfactory bulb of Sprague Dawley rats at 3, 12, 24, 27, and 30 months of age (Hinds and McNelly, 1977). They also assessed the number of mitral cells and the volume of their nuclei, cell bodies, and dendritic trees, as well as the total length and mean cross-sectional area of the associated dendrites. Until the age of 24 months, a ~50% developmental increase in the volumes of each of the layers was observed, as was a doubling in the size of the volumes of the cell bodies and dendritic trees of the mitral cells. From 24 to 30 months, the volume of olfactory bulb layers decreased. Subsequently, the number of mitral cells also decreased. Although the total volume of mitral cell dendritic trees declined slightly from 24 to 27 months, the volume of individual mitral cell dendritic trees, as well as cell body and nuclear size, increased, presumably reflecting compensation for the decrease in mitral cell numbers.

In a subsequent study, these general findings were replicated in the Charles River rat strain (Hinds and McNelly, 1981), save for a lack of a decline in mitral cell number in the older animals. Concurrent assessment was also made of alterations in the olfactory epithelium. After 18 months, olfactory bulb volumes declined and, after 24 months, decreases in the average volumes of the mitral cell bodies and the glomerular dendrites were observed. A comparison of regression lines for changes in number of olfactory receptors on the septum with that of the size of mitral cell bodies suggested that the decline in receptor number began several months before the decline in mitral cell size. This implied that the bulbar changes were in response to the epithelial changes. In the remaining olfactory receptor cells, an increase in the number of synapses per cell within the glomeruli occurred in the oldest rats evaluated, implying compensatory responses.

Age-related changes in the volume of human olfactory bulbs have also been documented *in vivo* using magnetic resonance imaging (MRI) (Yousem et al., 1998; Buschhuter et al., 2008). However, such decrements are not specific to aging, are variable, and are plastic to some degree. Thus, olfactory bulb volume is reduced in cigarette smokers (Schriever et al., 2013) and in those with a number of neurological diseases or other disorders (Yousem et al., 1995b). These include acute depression (Negoias et al., 2010), Alzheimer's disease (Thomann et al., 2009), childhood abuse (Croy et al., 2013), chronic sinusitis (Rombaux et al., 2008), congential anosmia with and without Kallmann syndrome (Yousem et al., 1993, 1996; Abolmaali et al., 2002; Koenigkam-Santos et al., 2011; Levy et al., 2013), epilepsy (Hummel et al., 2012), head trauma (Yousem et al., 1995a; Doty et al., 1997; Landis et al., 2005; Jiang et al., 2009), multiple sclerosis (Goektas et al., 2011; Schmidt et al., 2011), Parkinson's disease (Wang et al., 2011b; Brodoehl et al., 2012), polyposis (Herzallah et al., 2013), schizophrenia (Turetsky et al., 2003; Nguyen et al., 2011), and prior upper respiratory infections associated with chronic smell loss (Rombaux et al., 2009). Such studies strongly suggest that olfactory bulb volume is a marker for olfactory function in general (Yousem et al., 1998; Turetsky et al., 2003; Buschhuter et al., 2008; Haehner et al., 2008; Hummel et al., 2011; Rombaux et al., 2012). Evidence of plasticity comes from observations that over time the shrinkage of olfactory bulbs in humans due to rhinosinusitis can be reversed as a result of treatment (Gudziol et al., 2009) and that rodent intrabulbar circuitry can recover from occlusion after reinstating nasal patency (Cummings and Belluscio, 2010).

### Changes in central brain regions involved in olfactory processing

It is widely appreciated that aging is accompanied by decreased brain weight, cortical thickness, white matter integrity, and transmitter activity, and increased neuronal vulnerability, including early changes within brain structures associated with olfactory system processing (Kemper, 1984). Among such changes are disproportionate decrements in the volume of the hippocampus, amygdala, piriform cortex, anterior olfactory nucleus, and frontal poles of the brain. In a cohort of non-demented subjects ranging in age from 51 to 77 years, Segura et al. (2013) found that UPSIT scores were significantly correlated with the volume of the right amygdala and bilaterally with the volume of gray matter in the perirhinal and entorhinal cortices. Such scores were also inversely correlated with cortical thickness in the postcentral gyrus and with fractional anisotropy and mean diffusivity levels in the splenum of the corpus callosum and the superior longitudinal fasciculi.

A number of age-related neurodegenerative disease pathologies, including abnormal deposits of tau and α-synuclein, have been associated with olfactory dysfunction in older non-demented persons, suggesting that some age-related alterations may reflect “pre-clinical” neurodegenerative disease. For example, in a longitudinal clinicopathological study of 122 non-demented subjects, Wilson et al. (2007a) found inverse correlations between B-SIT scores obtained before death and the post-mortem density of neurofibrillary tangles in the entorhinal cortex, the CA1 subfield of the hippocampus, and the subiculum. Similar associations were found by this group between pre-mortem B-SIT scores and post-mortem measures of Lewy bodies within limbic and cortical brain regions (Wilson et al., 2011b), leading the authors to conclude that olfactory function is impaired in Lewy body disease even in otherwise asymptomatic individuals.

Persons with mild cognitive impairment (MCI) who convert to AD typically have more smell loss than MCI patients who do not convert (Croy et al., 2009). In AD, tau-related neurofibrillary pathology seems to be more closely linked to olfactory dysfunction than β-amyloid plaque pathology. Thus, some studies find no direct associations between the AD-related decrement in odor identification ability and brain β-amyloid, as measured by PET imaging of Pittsburgh compound B, an *in vivo* marker of brain amyloid levels (Bahar-Fuchs et al., 2010). This observation is consistent with post-mortem studies which, after controlling for the adverse influences of tau, find no strong associations between pre-mortem olfactory function and post-mortem levels of β-amyloid in olfactory eloquent brain regions of older individuals (Wilson et al., 2007a). It is noteworthy that anosmia, *per se*, is correlated with wide spread changes in gray matter within olfaction-related structures, including the piriform cortex, insular cortex, OFC, medial prefrontal cortex, hippocampus, parahippocampal gyrus, supramarginal gyrus, nucleus accumbens, subcallosal gyrus, and the medial and dorsolateral prefrontal cortices (Bitter et al., 2010).

**Figure 13:**
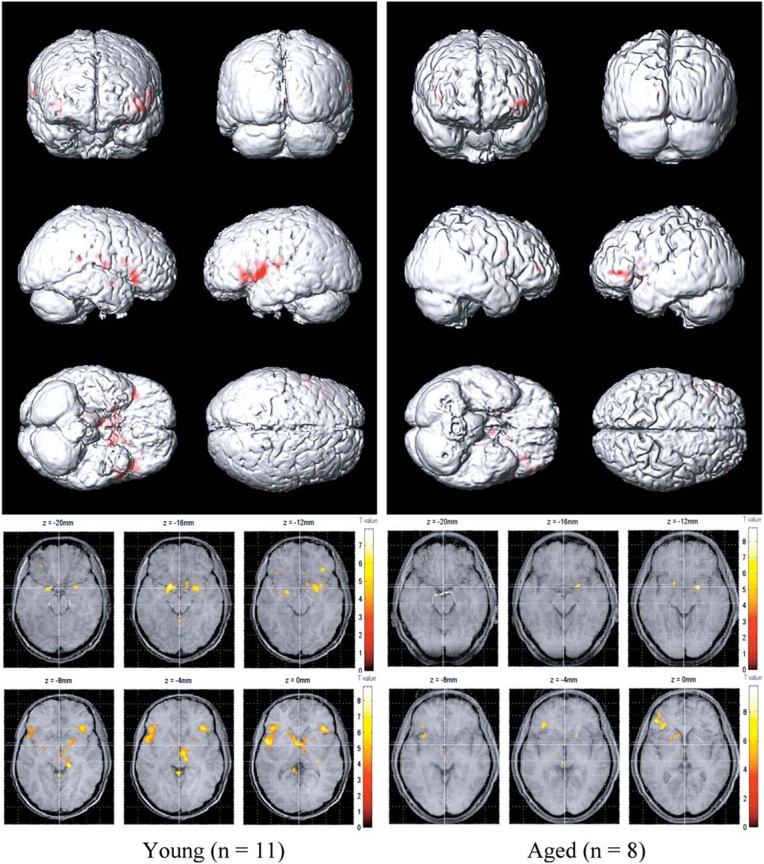
**Olfactory functional magnetic resonance imaging (fMRI) activation maps from 11 younger (left; mean age = 23.9 years) and 8 older (right; mean age = 66.4 years) persons to lavender and spearmint odors.** Note greater activation in the younger subjects. From Wang et al. (2005), with permission. Copyright©2005, Gerontological Society of America.

Functional imaging studies, such as those employing fMRI and positron emission tomography (PET), also demonstrate age-related changes in the processing of olfactory information, as reflected by decrements in odor-induced activation in central olfactory pathways. It should be noted, however, that such decrements need not be indicative of the locus of dysfunction. This is because the activity of a given central brain region often depends upon input from other brain regions that themselves may be compromised. Nevertheless, such imaging does represent the overall functioning of the system. Yousem et al., in a pioneering fMRI study, found that odors activated fewer voxels within the right inferior frontal and left and right superior frontal and perisylvian zones in old than in young persons (Yousem et al., 1999). Subsequently, Suzuki and his associates noted less fMRI odor-induced activation in 6 older persons than in 6 younger persons during an odor discrimination task in a region within the left orbital pole (Suzuki et al., 2001). Wang et al. (2005) found age-related decreases in activation in structures comprising the primary olfactory cortex, most notably in the right amygdala and piriform and periamygdaloid cortices (Figure 13). These investigators chose subjects whose UPSIT scores were within the normal age-adjusted range, although slightly lower scores were evident in the 11 young subjects (mean age = 23.9) than in the 8 older subjects (mean age = 66.4); respective UPSIT means = 37.3 and 34.1, *p* = 0.0004. More recently, Wong et al. (2010) found that a measure of nigrostriatal denervation in healthy elderly persons over the age 60 years, as determined by PET imaging of the brain dopamine transporter (DAT), was significantly correlated with UPSIT scores, suggesting that age-related declines in nigrostriatal function may account, in part, for age-related losses in smell ability.

### Neurochemical changes in the brain

It is well established that age-related changes occur in numerous enzyme, neurotransmitter, and neuromodulator systems within the brain. In many cases, the largest age-related decline occurs before the age of 60 years, as exemplified by enzymes involved in the synthesis of GABA (glutamate decarboxylase), acetylcholine (choline acetyltransferase), and both norepinephrine and dopamine (tyrosine hydroxylase) (Selkoe and Kosik, 1984). Hence, significant neurochemical changes are likely present prior to the onset of neuropathology and cognitive and motor phenotypes associated such age-related diseases as AD and PD. This implies that some such changes may “prime” the organism or lower the threshold for adverse influences from neural insults, mutations, and other deleterious factors in the elderly and could be, in fact, a critical substrate for the so-called “preclinical” stages of some age-related neurodegenerative diseases. Importantly, such neurochemical changes may be region specific, preferentially involving, for example, limbic structures early in the aging process (Strong, 1998). Imaging studies suggest that binding sites for a number of neurotransmitters are significantly decreased in the brains of older persons (Dewey et al., 1990; Rosier et al., 1996; Volkow et al., 2000).

While several age-related neurotransmitter deficiencies may contribute to the olfactory loss observed in elderly persons, one system stands out as being particularly prepotent—the cholinergic system. Acetylcholine is intimately involved in the modulation of olfactory function, such as increasing contrast and synchronization of odor-induced activity from the bulb to the piriform cortex and facilitating attention, odor learning, memory, and cortical plasticity (de Almeida et al., 2013). Cholinergic projections reach all sectors of the olfactory system from origins within the medial septum, the nucleus basalis of Meynert, and the horizontal and vertical diagonal band of Broca (Schliebs and Arendt, 2011). Patients with MCI exhibit olfactory deficits and cholinergic dysfunction prior to the onset of AD—dysfunction which is unaccompanied by significant cell loss (Schliebs and Arendt, 2011). Interestingly, cholinergic neurons directly modulate neural activity within the olfactory bulb and tonically inhibit, along with some other neurotransmitters, the activity of microglial cells critical for immune responses to brain damage and foreign agents (Doty, 2012a). When such modulation is significantly perturbed, the release of inhibition on the microglial cells can occur, resulting in the secretion of inflammatory mediators and other factors which, in extreme instances, can be deleterious to neurons (Tang et al., 2007; Lalancette-Hebert et al., 2009).

It is noteworthy that the relative magnitude of olfactory deficits of a number of neurodegenerative and neurodevelopmental diseases appears to be associated with the relative damage to the basal cholinergic system. Such disorders include AD, PD, Down syndrome, Parkinson-Dementia complex of Guam, Korsakoff syndrome, amyotrophic lateral sclerosis, schizophrenia, and progressive supranuclear palsy (for olfactory test scores, see, e.g., Mair et al., 1986; Doty et al., 1987, 1988, 1991, 1993; Kopala et al., 1994; Sajjadian et al., 1994; Wenning et al., 1995; McKeown et al., 1996; for quantitative assessments of basal cholinergic cell losses or volumes, see Arendt et al., 1983; Nakano and Hirano, 1983, 1984; Casanova et al., 1985; Rogers et al., 1985; Vogels et al., 1990; Yoshida et al., 1992; Kasashima and Oda, 2003). Age-related damage to the nucleus basalis has also been observed, although its magnitude is not generally as marked as that seen in AD and PD.

## Conclusion

This review addressed the functional and pathophysiological changes that occur in the human olfactory system as a result of age. Basic information about the anatomy, physiology, and measurement of this primary sensory system was provided, along with a general overview of the nature of age-related changes that occur in olfactory perception. Numerous factors that likely contribute to such changes were assessed, including changes in autonomic control of nasal engorgement, increased propensity for nasal disease, cumulative damage to the olfactory epithelium from environmental insults, decrements in protective metabolizing enzymes in the olfactory mucosa, occlusion of the foramina of the cribriform plate, loss of selectivity of olfactory receptor neurons to odorants, changes in neurotransmitter and neuromodulator systems, and neuropathological processes such as the expression of aberrant proteins associated with such neurodegenerative diseases as AD and PD. As apparent from the research examined in this review, it is likely that there are multiple determinants of the olfactory loss of older persons, although the relative importance of each is yet to be established.

## Author contributions

Dr. Doty wrote the first draft of the manuscript. Dr. Kamath contributed to the literature review and to the writing of two subsequent drafts. Both authors were involved in the writing of the final manuscript.

### Conflict of interest statement

Dr. Doty is the President of, and major shareholder in, Sensonics International, a corporation that manufactures and distributes chemosensory test equipment and products, including the commercial version of the University of Pennsylvania Smell Identification Test (UPSIT) and a number of its clones. Dr. Kamath declares no commercial or financial relationships that could be construed as a potential conflict of interest.
